# The farther the better: Investigating how distance from human self affects the propensity of a peptide to be presented on cell surface by MHC class I molecules, the case of Trypanosoma cruzi

**DOI:** 10.1371/journal.pone.0243285

**Published:** 2020-12-07

**Authors:** Davide Vergni, Rosanna Gaudio, Daniele Santoni

**Affiliations:** 1 Istituto per le Applicazioni del Calcolo “Mauro Picone” - CNR, Rome, Italy; 2 Department of Biology, University Tor Vergata, Rome, Italy; 3 Istituto di Analisi dei Sistemi ed Informatica “Antonio Ruberti” - CNR, Rome, Italy; University of Pittsburgh, UNITED STATES

## Abstract

More than twenty years ago the reverse vaccinology paradigm came to light trying to design new vaccines based on the analysis of genomic information in order to select those pathogen peptides able to trigger an immune response. In this context, focusing on the proteome of *Trypanosoma cruzi*, we investigated the link between the probabilities for pathogen peptides to be presented on a cell surface and their distance from human self. We found a reasonable but, as far as we know, undiscovered property: the farther the distance between a peptide and the human-self the higher the probability for that peptide to be presented on a cell surface. We also found that the most distant peptides from human self bind, on average, a broader collection of HLAs than expected, implying a potential immunological role in a large portion of individuals. Finally, introducing a novel quantitative indicator for a peptide to measure its potential immunological role, we proposed a pool of peptides that could be potential epitopes and that can be suitable for experimental testing. The software to compute peptide classes according to the distance from human self is free available at http://www.iasi.cnr.it/~dsantoni/nullomers.

## 1 Introduction

*Trypanosoma cruzi*, a protozoan parasite belonging to the phylum Euglenozoa, is the etiologic agent of Chagas disease, a tropical pathology also known as american trypanosomiasis. [1] According to recent statistics it affects more than eight million people and it is responsible for more than eight thousand casualties every year in Central and South America (https://www.who.int/chagas/epidemiology/en/). Although many efforts have been done by the scientific community to face up this emergency a vaccine is not still available for human [2, 3]. Several approaches have been proposed to build a vaccine, focusing on secreted or membrane associated proteins such as trans-sialidase family [4–6] and several studies tested promising epitopes in mice [7] and dogs [8]. Others studies [9, 10] proposed a computational approach based on whole genome screening, according to the reverse vaccinology paradigm [11, 12].

In the postgenomic era the wide availability of genomic data and the development of accurate tools make possible the use of bioinformatics for a broader evaluation of potential epitopes. Several papers focused on the computational identification of potential epitopes for vaccine design [13–18]. Usually those works considered different features that peptides have to show to be epitopes, including epitope conservancy analysis, epitope toxicity prediction, binding stability and in some cases molecular docking with specific Human Leukocyte Antigen (HLA). The focal point anyway is the prediction of binding to the Major Histocompatibility Complex (MHC) estimated through validated and solid predictive algorithm (see the review of Schirle and colleagues for reference [19]). In particular He and colleagues developed a Web-Based Vaccine Design tool called Vaxign, applied to more than 70 genomes [20]. Vaxign pipeline takes into consideration several features including protein subcellular location, transmembrane helices, adhesin probability, conservation to human and/or mouse proteins, sequence exclusion from genomes of nonpathogenic strains, and epitope binding to MHC class I and class II.

According to the self/non-self paradigm, the overwhelming majority of microorganisms peptides are not shared with human and only a small percentage of peptides are in common (approximately 0.2% [21])). Therefore the most part of pathogen peptides can be defined as *nullomers* of human proteome. The word *nullomer*, indicating an absent word for a given reference sequence, has been introduced for the first time by Hampikian and Andersen in 2007 [22]. To date, there are several works dealing with the study of absent words in biological sequences, focusing both on DNA [23–25] and protein sequences [26, 27]. Vergni and Santoni [28] in 2016 introduced an extension of nullomers, namely high order nullomers, i.e., absent words whose mutated sequences are still absent. In the present paper nullomer classes have been defined by introducing a distance between a given peptide and human self in terms of the minimal number of mutation steps needed to transform the peptide into a human one.

Focusing on the proteome of *Trypanosoma cruzi*, the relationship between the likelihood of a peptide to be presented on the cell surface and its distance from human self has been investigated. This study ideally follows the work of Santoni [29] where it has been highlighted, considering the proteomes of Human Immunodeficiency Virus type 1 (HIV1) and Human herpes simplex virus 1 (HHV1), that peptides far from human self more than three mutation steps show a strong propensity to bind the MHC class I molecules. MHC class I molecules (MHC-I) play a focal role in adaptive immune system. They are highly polymorphic proteins able to bind antigenic peptides and present them to T cells. The complex formed by an immunogenic peptide and an element in MHC-I is exposed on nucleated cells surface and can be recognized by cytotoxic CD8+ T cells activating them and triggering the immune response. Moreover, in the wake of the medical crisis caused by the covid-19 pandemic, a short report in which similar methodologies have been applied to the search of potential epitopes of the SARS-CoV-2 virus has been proposed [30]. In this work we deepen and extend those results detailing how the use of high order nullomers can be influential in the search for potential epitopes. The idea is to partition exogenous peptides in different nullomer classes through their distance from human proteome, discriminating between peptides that can be considered farther or closer to human self. This approach can contribute to extend the self/non-self paradigm by associating to farther peptide a strong non-self status while to closer peptide a weak non-self status. By using tested and accurate bioinformatics tools (netMHC [31–34] and NetCTL [34–37], for the prediction of i) Proteasome Cleavage (CLE), ii) Transporter Associated with Antigen Processing (TAP) and iii) MHC-I binding) we are able to reproduce in silico the whole pipeline that brings a peptide to be presented on the cell surface. A high co-evolution among those three steps is highlighted, particularly for farthest from human peptides, extending already known results [21] to all available HLAs. We also show even more strong co-evolution factors when considering the most distant peptides from human self. Moreover we discuss how distant peptides from human self tend to bind a higher number of HLAs than expected, so that they can trigger an immune response in a large portion of population. Using a novel methodology we identified two different sets of peptides that could be considered potential epitopes and which may be suitable for experimental tests in order to validate their immunogenicity. Finally information content analysis, performed on the considered pool of promising peptides, revealed an evident aminoacid pattern and a minor sequence complexity. This implied strong sequence constraints for the best promising peptides.

## 2 Methods

### 2.1 Proteomes

The proteome of Homo sapiens (HSA) GRCh38, has been downloaded from Ensembl web site (http://ftp.ensembl.org/pub/current_fasta/homo_sapiens/pep/). Available strain protein sequences of *Trypanosoma cruzi* (TC)—44,512 sequences (3703 from TC model organism—NCBI taxonomy 5693, 10213 from TC *marinkellei*—NCBI taxonomy 85056, 19244 from TC *CL Brener*—NCBI taxonomy 353153 and 11352 from TC *Dm28c*—NCBI taxonomy 1416333)—have been downloaded from UNIPROT site (http://www.uniprot.org/uniprot/). Ad hoc python scripts have been designed in order to extract all unique 9-mers from the sequences of the considered organisms, removing all those 9-mers containing other than the 20 standard aminoacids. We formally define the following peptide sets:

- *HSA*^9^: unique 9-mers occurring in reference human proteome;
- *TC*^9^: unique 9-mers occurring in available *Trypanosoma cruzi* proteins.

In the following, for the sake of simplicity, we will omit the superscript 9, since we will always refer to 9-mers. We will also refer to *HSA* as human self, even if peptide size is limited to 9.

### 2.2 Peptide classes: Distance from human self

Let *p* ≡ (*p*_1_, *p*_2_, ⋯, *p*_9_) and *q* ≡ (*q*_1_, *q*_2_, ⋯, *q*_9_) be two 9-mers, *p* ∈ *TC* and *q* ∈ *HSA*, and *B*(*i*) be the mutation indicator function that assumes the value 1 if at position *i* (*i* = 1, .., 9) the aminoacids *p*_*i*_ and *q*_*i*_ are different

$$\begin{array}{c} B\left(i\right)=\{\begin{array}{cc} 0 & p_{i}=q_{i}\\ 1 & p_{i}\neq q_{i} \end{array}\;\;. \end{array}$$ (1)

Using the function *B*(*i*) it is easy to define the distance between *p* and *q* in terms of number of mutations between them as:

$$\begin{array}{c} D\left(p,q\right)=\sum_{i=1}^{9}B\left(i\right)\;. \end{array}$$ (2)

*D*(*p*, *q*) is the Hamming distance between the two peptides. Using the distance *D* it is possible to define the distance between a peptide *p* ∈ *TC* and the entire human self *HSA* as follows:

$$\begin{array}{c} M\left(p\right)=\min_{q\in HSA}\;\left\{D\left(p,q\right)\right\} \end{array}$$ (3)

By definition if *M*(*p*) is equal to *m* no human 9-mer can be obtain from *p* by mutating a number of aminocids smaller than *m*. According to the distance *M* we define different disjoint subsets of *TC*:

- the common class, *C*, as:
  $$\begin{array}{c} C=\{p\in TC\;|\;M(p)=0\} \end{array}$$ (4)
  i.e., the subset of TC peptide in common with HSA;
- a first class of absent peptides, *W*_1_, as
  $$\begin{array}{c} W_{1}=\left\{p\in TC\;|\;M\left(p\right)=1\right\} \end{array}$$ (5)
  containing peptides that are not present in *HSA* but that can be changed in at least one human 9-mer with a single mutation step;
- a second class of absent peptides, *W*_2_, as
  $$\begin{array}{c} W_{2}=\left\{p\in TC\;|\;M\left(p\right)=2\right\} \end{array}$$ (6)
  containing peptides that are not present in *HSA*, as well as every 9-mer obtained by single mutation of them, but they can be changed in at least one human 9-mer with two mutation steps;
- generalizing, an *m*-th class of absent peptides, *W*_*m*_, as
  $$\begin{array}{c} W_{m}=\left\{p\in TC\;|\;M\left(p\right)=m\right\}. \end{array}$$ (7)
  It follows that a peptide belonging to class *W*_*m*_ needs *m* mutation steps (not less than *m*) in order to be changed in at least one human peptide.

In the following we will also refer to peptides belonging to set *W*_1_ as nullomers, i.e., absent words in the sequence HSA, while to peptides belonging to class *W*_*i*_ (with *i* > 1) as high order nullomers.

Those classes naturally induce a partition of *TC* in disjoint subsets:

$$\begin{array}{c} TC=C\cup W_{1}\cup W_{2}\cup ...\cup W_{9}\;\;\\ \text{where}\;C\cap W_{i}=\emptyset \;\;\;\;\forall i\;\;\;\;\text{and}\;\;\;\;W_{i}\cap W_{j}=\emptyset \;\;\;\;\forall i\neq j\;. \end{array}$$ (8)

Unlike [28] in which nullomers and high order nullomers are gathered in non-disjoint sets, in this work we preferred to use disjoint classes of nullomers, *W*_*i*_, in order to characterize in a clearer way the differences among peptides with different distance from human self.

### 2.3 Prediction softwares

#### 2.3.1 Peptide-MHC-I interaction prediction score: NetMHC

The software NetMHC (version 4.0) [31] has been used to predict the interaction of peptides with the MHC-I complex in terms of binding scores, taking into account 81 available different HLAs (36 class A, 34 class B, 10 class C and 1 class E). NetMHC provides for every 9-mer *p* ∈ *TC* and for any given HLA *h*_*i*_ a binding score, that we call *α*_*i*_(*p*) for *i* = 1, 2, .., 81.

According to NetMHC software three possible interval scores are defined:

- NB—No Bind: score higher than 2;
- WB—Weak Bind: score higher than 0.5 and equal or smaller than 2;
- SB—Strong Bind: score equal or smaller than 0.5.

Since we are interested in those peptides that are likely to be presented on a cell surface, we focus on strong bind defining and indicator function *ϵ*_*i*_(*p*) as

$$\begin{array}{c} \epsilon_{i}\left(p\right)=\{\begin{array}{cc} 1 & \alpha_{i}\left(p\right)\leq 0.5\\ 0 & otherwise \end{array} \end{array}$$ (9)

for *i* = 1, 2, .., 81.

Considering the entire set of HLAs included in NetMHC, some useful properties associated to a given peptide can be defined as follows

- The best score function, *α*(*p*), namely the smallest score out of all the 81 ones
  $$\begin{array}{c} \alpha \left(p\right)=\min_{i=1,2,..,81}\left\{\alpha_{i}\left(p\right)\right\}\;\;; \end{array}$$ (10)
- The indicator function, *ϵ*(*p*), whose value is 1 if the peptide *p* strongly binds at least one HLA:
  $$\begin{array}{c} \epsilon \left(p\right)=\{\begin{array}{cc} 1 & \alpha (p)\leq 0.5\\ 0 & otherwise \end{array}\;\;; \end{array}$$ (11)
- The total number of HLAs, *N*(*p*), that strongly bind a peptide as
  $$\begin{array}{c} N\left(p\right)=\sum_{i=1}^{81}\epsilon_{i}\left(p\right)\;. \end{array}$$ (12)

#### 2.3.2 Proteasome cleavage and TAP transport prediction scores: NetCTL

The software NetCTL (version 1.2) [34–37] has been used to predict proteasome cleavage probability and TAP Transport scores associated to considered peptides. The cleavage prediction is provided in terms of probability, where 0 indicates no probability to be cleaved and 1 indicates certainty to be cleaved, while TAP Transport prediction is provided as a score, not mapped into a probability. According to [35] a significant threshold for the score was identified: it was assessed that only 1.5% of epitopes have a score smaller than −1. We associated to every considered peptide, *p*, the related TAP Transport score, *TAP*(*p*) and the best proteasome cleavage score (since the cleavage site changes depending on the sequence context) *CLE*(*p*).

### 2.4 Statistical analysis

In order to statistically assess the tendency of peptides farther from human self to strongly bind MHC-I, hypergeometric tests were applied to perform enrichment analysis [38]. In other words we used hypergeometric test to evaluate whether the number of strong bind peptides in the classes of 9-mers farther from human self was significantly higher than expected (with respect to the number of strong bind peptides in the whole set of *TC* 9-mers). Hypergeometric tests were run through R scripts, providing the P-value of the test.

## 3 Results

We analyzed peptide classes defined above (*C*, *W*_1_, *W*_2_, …) to investigate whether the distance from human self, that is the criterion we used to partition peptides in classes, affects the likelihood of peptides to be presented on a cell surface. In the next subsections we will firstly compare binding affinity of peptide classes to MHC-I showing that peptides with higher distance from human self have an average binding score smaller than peptides closer to human self and that they tend to strongly bind a higher number of HLAs. We will also take into consideration proteasome cleavage and TAP transport as further selective steps to identify those peptide that are more likely to be presented on a cell surface. Finally we will provide two sets of peptides, related to TC and *W*_4_, respectively, with the highest probability to be presented on a cell surface showing how the best promising peptides present regularities in their sequence according to a Shannon entropy measure.

### 3.1 Peptide classes and MHC-I binding scores

First of all we built peptide classes defined above, *C*, *W*_1_, *W*_2_, *W*_3_ and *W*_4_, according to the distance function *M*(*p*).

No peptide resulted to be *W*5, in other words four steps mutations are sufficient to obtain from every peptide in *TC* at least one peptide in *HSA*. Such a result is not surprising considering the huge number of peptides that can be obtained by permitting up to four mutations in the set of TC containing almost nine millions of peptides. The number and percentage of peptides in each class are reported in Table 1.

**Table 1:** Number and percentage of unique peptides of Trypanosoma cruzi (*TC*) are reported together with the partition in disjoint classes *C*, *W*_1_, *W*_2_, *W*_3_ and *W*_4_.

	*TC*	*C*	*W*_1_	*W*_2_	*W*_3_	*W*_4_
# peptides	8,937,165	19,966	303,662	4,663,994	3,939,218	10,325
Percentage	100	0.22	3.40	52.19	44.08	0.11

**Fig 1:**
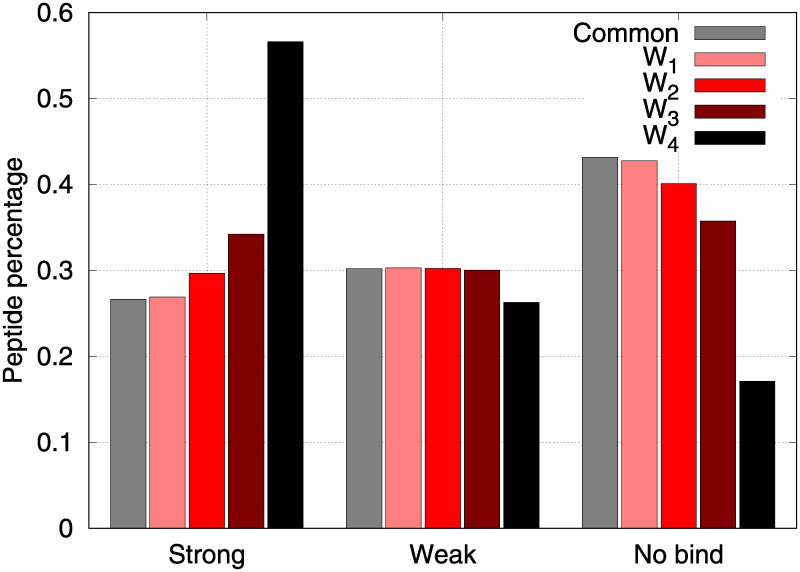
Percentages of MHC-I bound types (no bind, weak bind and strong bind) in the various peptide classes (*C*, *W*_1_, *W*_2_, *W*_3_ and *W*_4_) for Trypanosoma cruzi (*TC*) unique peptides.

In Table 2 for each class (*C*, *W*_1_, *W*_2_, *W*_3_ and *W*_4_) the numbers and the percentages of strong, weak an no bind peptides are reported while Fig 1 shows the composition of the classes in terms of strong bind (SB), weak bind (WB) and no bind (NB) expressed as percentages. The histograms clearly show that *W*_4_ (black bars) has a peculiar behaviour with respect to other classes: the composition percentage are directly correlated to the binding class, i.e. the stronger the bind the higher the percentage of *W*_4_ peptides in that class, while there is an inverse correlation for sets *C* and *W*1 and there is no clear correlation for sets *W*_2_ and *W*_3_.

**Table 2:** Number (percentages with respect to the total number of peptides in the class) of strong, weak and no bind peptides for peptide classes *C*, *W*_1_, *W*_2_, *W*_3_ and *W*_4_.

	*SB*	*WB*	*NB*
All	2,824,849 (32%)	2,693,897(30%)	3,418,419(38%)
C	5,317(27%)	6,029(30%)	8,620(43%)
W1	81,738(27%)	92,048(30%)	129,876(43%)
W2	1,383,655(30%)	1,410,721(30%)	1,869,618(40%)
W3	1,348,296(34%)	1,182,385(30%)	1,408357(36%)
W4	5,843(57%)	2,714(26%)	1,768(17%)

In other words peptides farther from human self show a clear tendency to strongly bind HLAs, observing on average a significantly higher strong bind percentage with respect to other peptides. As can be observed in Table 2 the percentages of strong bind peptides is significantly higher than expected in those classes (W3 and in particular W4) whose peptides are farther from human self. On the contrary, the number (and the percentage) of no bind peptides is significantly higher than expected in those classes (C, W1 and W2) whose peptides are closer to self. Hypergeometric tests were performed to statistically evaluate whether peptide classes (C, W1, W2, W3 and W4) were significantly enriched in strong bind peptides with respect the whole set of *TC* 9-mers. Obtained test results confirmed that peptides farther from human self (W3 and W4) show a significant tendency to strongly bind MHC class I complex while peptides close to human self (C, W1 and W2) show a number of strong bind 9-mers close to expected. Enrichment analysis provided very significant P-values, practically 0 (*P*_*value*_ ≪ 10^−10^) for W4 and W3, and P-values very close to 1 (*P*_*value*_ > 0.99) for C, W1 and W2.

In order to better characterize peptides belonging to the different sets, we studied two other quantities previously introduced in section 2.3.1, i.e., the best score, *α*(*p*), and the numbers of HLAs a given peptide strongly binds, *N*(*p*).

**Fig 2:**
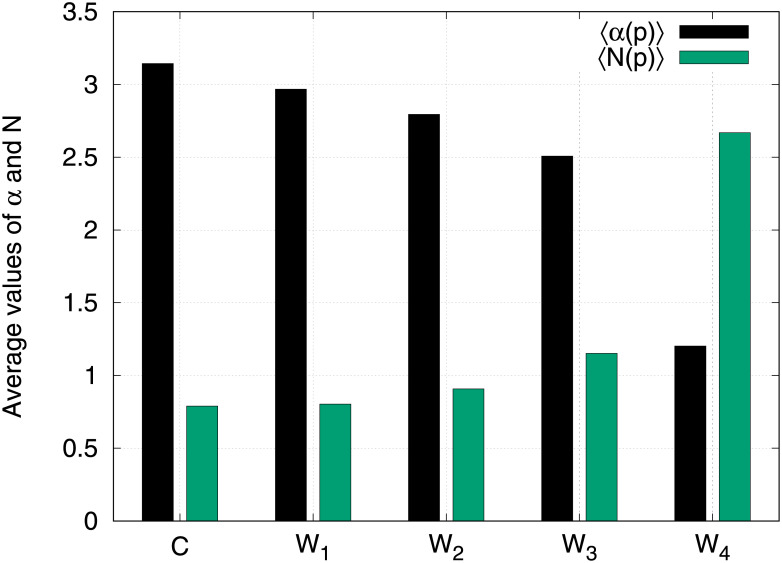
The average of both best score function, *α*(*p*), and numbers of HLAs a given peptide strongly binds, *N*(*p*), are reported for each peptide classes.

In Fig 2 it is reported the average of those quantities in the different sets, i.e. 〈*α*(*p*)〉_*p*∈*P*_ and 〈*N*(*p*)〉_*p* ∈ *P*_ where *P* = (*C*, *W*_1_, *W*_2_, *W*_3_, *W*_4_). Fig 2 clearly shows that peptides belonging to *W*_4_ set have on average a lower best score than other classes and they strongly bind an average number of HLAs that is more than three times higher than those of *C*, *W*_1_ and *W*_2_ (2.7 against 0.8, 0.8 and 0.9) and more than two times higher than that of *W*_3_ (2.7 against 1.18). This last finding is particularly interesting in the view of selecting peptides able to trigger an immune response since it highlights that peptides far from human not only tend to have higher binding probability but they can also strongly bind a broader collection of HLAs, so that they can play an immunological role in a large portion of individuals.

**Fig 3:**
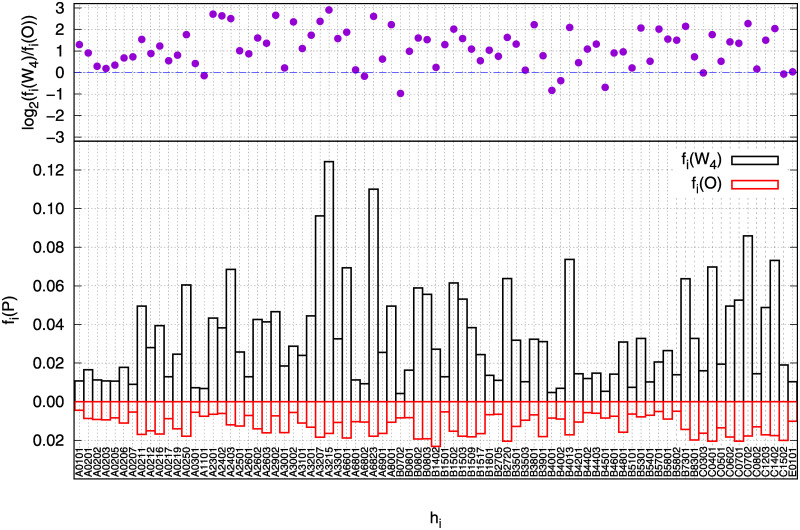
The percentage of strong binding peptides for sets W4 (black bars) and O = W1 U W2 U W3 (red bars) are reported in the lower panel for each HLA considered in the present paper. The graph shows that in the W4 set there are a much larger number of strong binding peptides than in the O set. In the upper panel the logarithmic ratio between the two percentages are shown in order to evidentiate the difference in amplitude of the percentages.

The percentages of SB peptides for each HLA have been computed for *W*_4_ and for *W*_1_ ∪ *W*_2_ ∪ *W*_3_ ≡ *O* (Others), and reported in Fig 3. To be more specific, we computed for classes *P* = (*O*, *W*_4_) the quantity

$$\begin{array}{c} f_{i}\left(P\right)=\frac{\sum_{p\in P}\epsilon_{i}\left(p\right)}{\#(P)} \end{array}$$ (13)

where the numerator accounts for the number of peptides in the class *P* that strongly bind the HLA *h*_*i*_ and the denominator #(*P*) indicates the number of elements in the class *P*.

As one can observe in the lower panel of Fig 3, the percentages related to the 81 HLAs for *W*_4_ (black bars) are clearly higher (with a few exceptions) with respect to the ones of *O* (red bars). The highest percentages for *W*_4_ are reached by HLA-A3215 (*f*_*A*3215_(*W*_4_) = 0.12, i.e., 1284 SB peptides out of 10235, the total number of peptides in *W*_4_), HLA-A6823 (*f*_*A*6823_(*W*_4_) = 0.11, i.e. 1137 SB peptides) and HLA-A3207 (*f*_*A*3207_(*W*_4_) = 0.10, i.e., 993 SB peptides). On the other side the highest percentages for *O* reach a bit more than 0.02. In the upper panel of the figure the log_2_ ratios between *W*_4_ and *O* percentages for each HLA are reported, i.e. log_2_(*f*_*i*_(*W*_4_)/*f*_*i*_(*O*)), to have an at-a-glance view of the whole scenario. Once again also Fig 3 shows that, on average, peptides belonging to *W*_4_ class bind much more HLAs than peptides belonging to other classes.

### 3.2 *In silico* pipeline: Proteasome cleavage—TAP transport—MHC-I binding

As reported in Materials and Methods by means of NetCTL package, the score associated to the probabilities that a given 9-mer can be obtained by proteasome cleavage and can be transported by TAP were computed. In this way we aimed at reproducing *in silico* the whole pipeline made of three steps that brings a peptide to be presented on cell surface: Proteasome cleavage, TAP Transport and MHC-I binding.

In order to investigate the dependence of the three steps, we set cutoff scores (≥0.5 for cleavage, ≥0 for TAP and ≤0.5 for MHC-I) and we computed the numbers of peptides predicted to pass the different steps (a similar analysis limited to only two different HLAs has been presented in [21]). Results are reported in Tables 3 and 4 for *W* (where *W* = *W*_1_ ∪ *W*_2_ ∪ *W*_3_ ∪ *W*_4_) and *W*_4_ classes, respectively.

**Table 3:** Numbers of unique peptides and percentages for Trypanosoma cruzi related to selection steps: Proteasome cleavage (CLE) higher than or equal to 0.5, TAP transport (TAP) higher than or equal to 0 and MHC-I binding (MHC-I SB) smaller than or equal to 0.5 for *W* = *W*_1_ ∪ *W*_2_ ∪ *W*_3_ ∪ *W*_4_.

*W*	CLE (%)	TAP (%)	MHC-I SB (%)
8,917,199	2,903,390 (32)	2,388,082 (82)	1,603,821 (67)
8,917,199	—	3,985,392 (45)	2,171,288 (54)
8,917,199	—	—	2,819,532 (32)

**Table 4:** Numbers of unique peptides and percentages for Trypanosoma cruzi related to selection steps: Proteasome cleavage (CLE) higher than or equal to 0.5, TAP transport (TAP) higher than or equal to 0 and MHC-I binding (MHC-I SB) smaller than or equal to 0.5 for *W*_4_.

*W*_4_	CLE (%)	TAP (%)	MHC-I SB (%)
10,325	3,217 (31)	2,903 (90)	2,563 (88)
10,325	—	5,994 (58)	4,526 (76)
10,325	—	—	5,843 (57)

In each row of the two tables we can observe the flow of the numbers (and percentages) of peptides passing the steps. From left to right the numbers and percentages are derived from the set of peptides related to the previous (left) non-empty cell. For example in the first row of Table 3, we considered all absent peptides of *TC*, *W*, and we selected among them those peptides with a cleavage score higher than 0.5 obtaining 2,903,390 ones (32%).

Starting from this set we then selected those peptides with a TAP score higher than 0 obtaining 2,388,082 ones (82% of already cleaved peptides). We finally applied MHC-I filter (at least one HLA with a binding score smaller or equal to 0.5), obtaining 1,603,821 peptides (67% of the already TAP transported). In the second row of Table 3 we started from the same set of peptides *W* but selected them with TAP threshold (jumping cleavage step) obtaining 3,985,392 peptide (45% of *W*), then we applied MHC-I filter obtaining 2,171,288 (54% of already TAP transported). In the last row we directly applied MHC-I filter obtaining 2,819,532 peptides (32% of *W*).

It is worth noting that the percentages occurring in the corresponding columns decrease (from top to bottom), meaning that the three steps are not independent on each other, and a co-evolutive pressure acted on them. In fact the percentage of cleaved peptides that are predicted to be TAP transported is around 82% while the percentage of a any peptides belonging to *W* predicted to be TAP transported is only 45%. The same holds for the MHC-I filter with respect to TAP selective step. By applying to any *W* peptide MHC-I filter we obtain a percentage of 32% while by applying the filter to already TAP transported peptides we obtain a significantly higher percentage of 54% that reaches 67% when applied to already cleaved and TAP transported peptides.

Table 4 was built in the same way than the previous one considering only *W*_4_ peptides. As can be observed the associated percentages are higher than those occurring in Table 3, suggesting a stronger co-evolutive pressure acting on peptides farther from human self. It is worth noting that 90% of peptides already cleaved are predicted to be TAP transported (with respect to 58% of all *W*_4_ peptides). 88% of *W*_4_ peptides strongly bind at least one HLA when proteasome cleavage and TAP transport filter steps are already applied instead of 76%, when only TAP filter is applied, and 57% when no previous filter is applied. It is important to point out that by changing the values of the cutoff scores the numerical values reported in the tables are modified but the highlighted cross-dependence among the selective steps still yields.

Finally, in order to test the reliability of the results, we carried out a specific study to investigate whether a strong bind between MHC-I and a given peptide predicted by netMHC was also a stable bind. We computed binding stability through NetMHCstab [39] choosing, among the list of available HLAs, the HLA B1501, that is the one with the highest number of peptides in the training set, i.e. the most reliable since other alleles were trained on smaller training peptide set. We selected 133 W4 peptides that were predicted through NetMHC to strongly bind B1501 HLA. 74 peptides out of 133 W4 were predicted through NetMHCstab to have a high binding stability with B1501 HLA and 124 a weak binding stability. This is a very good percentage with respect to average expected binding stability, guaranteeng robustness of our analysis.

### 3.3 Promising peptide

In this section we introduce a methodology able to select peptides with the highest probability to be presented on a cell surface according to previously discussed results. We considered the cleavage probability, fundamental first step for a peptide to be presented on a cell surface, and the number of HLAs that a peptide strongly binds. This last property is an important element for a peptide to have a good chance of being recognized by a large portion of population, since the greater the number of HLAs that strongly bind the peptide the larger the portion of population in which the peptide can trigger an immune response. As further discussed below, the TAP score is not determinant for the selection of the most promising peptides since its value is always well above -1 for those peptides having a high cleavage probability and a large number of HLAs they strongly bind.

Let *N*(*p*) be the number of HLAs that strongly bind a given peptide, *N*_*H*_ = 81 be the total number of considered HLAs, and *r* be the number of HLAs expressed by an individual, (typically *r* = 6 [40]), the “recognition probability”, i.e., the probability that at least one of the HLAs expressed by an individual strongly binds the peptide of interest, is given by the formula

R(p)=1-PHyper(k=0)=1-(N(p)0)(NH-N(p)r)(NHr)

where *P*_*Hyper*_(*k* = 0) is the probability (given by the hyper-geometric distribution) that taken *r* = 6 out of *N*_*H*_ = 81 HLAs none of them strongly binds the peptide.

The hyper-geometric distribution should be used when the probability for an individual to have a given HLA is independent from that HLA, but HLA alleles distribution is not uniform in the human population, moreover different human populations have different distributions of HLA. In this work, as a first approximation we used the hyper-geometric distribution. However, if one is interested in a specific population it is possible to consider their peculiar HLA frequency distribution to provide a more correct value of recognition probability. Considering as independent the cleavage probability, *CLE*(*p*), and the recognition probability, *R*(*p*), one can obtain the overall probability to be cleaved and recognized for a peptide by *l*(*p*) = *CLE*(*p*) ⋅ *R*(*p*). This approximation underestimates the overall probability (an indication of dependency between *CLE*(*p*) and *R*(*p*) arises from Table 3) therefore the obtained probability is certainly lower than the actual probability.

In Tables 5 and 6 are reported the best ranking peptides (with respect to measure *l*(*p*)) selected from the whole set TC and from the set *W*_4_, respectively.

**Table 5:** Ranking of the most promising peptides for Trypanosoma cruzi.

Peptide	CLE	TAP	N	R	is *W*_4_	l
FVYDFFYTL	0.9778	1.08	44	0.992836684	No	0.97079571
YVFEWFAAL	0.978	1.195	43	0.991493563	No	0.969680704
YMYSGGWTL	0.9753	1.252	43	0.991493563	No	0.967003672
FLFGFTYPL	0.9785	1.038	40	0.98614536	No	0.964943234
YMFAGTYSF	0.9781	2.757	40	0.98614536	No	0.964548776
YMMGWCYTL	0.976	1.253	41	0.988172868	Yes	0.964456719
FTFNYSAPL	0.9759	1.051	41	0.988172868	No	0.964357902
FMYDVLYAL	0.9758	1.119	41	0.988172868	No	0.964259085
FLFPFFYSL	0.9792	0.984	39	0.983836253	No	0.963372459
FMMGWCYTL	0.9769	1.2	40	0.98614536	Yes	0.963365402

In the first column the peptide sequence is reported. Second and third columns report the *CLE*(*p*) and *TAP*(*p*) scores, respectively. Fourth column reports the number of HLAs the given peptide strongly binds while in the fifth column the recognition probability, *R*(*p*), is reported. The sixth column indicates whether the peptide belongs or not to the *W*_4_ class. Finally, in the last column, the overall probability, *l*(*p*), is reported.

**Table 6:** Ranking of the most promising peptides selected from the set *W*_4_ for Trypanosoma cruzi.

Peptide	CLE	TAP	H	R	l
YMMGWCYTL	0.9760	1.2530	41	0.988172867919703	0.96445671908963
FMMGWCYTL	0.9769	1.2000	40	0.986145359563081	0.963365401757174
FMFGWCYTL	0.9780	1.1030	39	0.983836252823595	0.962191855261475
YMMGWCHTM	0.9717	0.4810	37	0.97824906852222	0.950564619883041
YMVGWCYTM	0.9741	0.4540	35	0.971138187077561	0.945985708032252
MMWEESMTM	0.9780	0.6150	33	0.962188008157362	0.9410198719779
FMIGWCYTM	0.9685	0.4480	35	0.971138187077561	0.940547334184618
YMHPISFKM	0.9775	0.3840	32	0.956911916272343	0.935381398156215
FTWPHYFYY	0.9749	2.8750	29	0.937269654125084	0.913744185806544
YIYWRHMWL	0.9727	1.2590	29	0.937269654125084	0.911682192567469

The labels in the columns are the same of Table 5.

**Fig 4:**
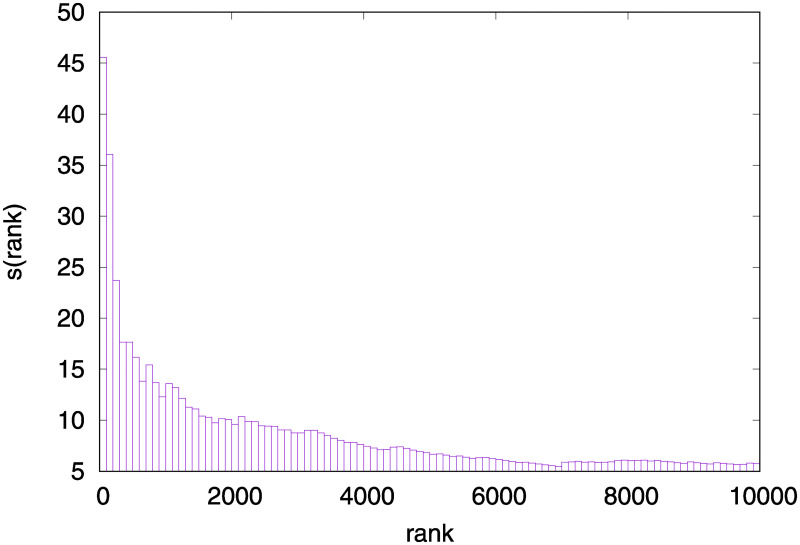
The ratio between the relative number of peptides *W*_4_ and not *W*_4_ for Trypanosoma cruzi up to position “rank” is shown.

Table 5 highlights there are only two *W*_4_ peptides in the first ten positions, in the sixth and tenth positions. Anyway, it is worth noting that the number of *W*_4_ peptides in the high ranking positions has to be weighted with respect to the total number of elements in the set. To be more specific, defining $N_{W_{4}}\left(rank\right)$ (and $N_{notW_{4}}\left(rank\right)$) as the number of *W*_4_ peptides (and not *W*_4_ peptides) in the ranking up to position “rank”, Fig 4 shows the plot of the ratio between $N_{W_{4}}\left(rank\right)$ and $N_{notW_{4}}\left(rank\right)$ normalized by the sizes of *W*_4_ and *notW*_4_ sets, respectively:

$$\begin{array}{c} s\left(rank\right)=\frac{N_{W_{4}}\left(rank\right)/\#\left(W_{4}\right)}{N_{notW_{4}}\left(rank\right)/\left(\#\left(TC\right)-\#\left(W_{4}\right)\right)}. \end{array}$$

This result again confirms *W*_4_ peptide propensity to be exposed and, in our opinion, they could be extremely interesting in the view of vaccine design. It can be hypothesized that *W*_4_ exposed peptides could have, on average, a higher number of potential antibodies able to recognize them due to the cross-reactivity mechanism. Cross-reactivity occurs when an antibody recognizes and binds antigens that are not specific for that antibody. In the original negative selection process, when an antibody targets a self peptide it is negatively selected and removed. Thus antibodies that target a peptide close to self-peptides, such as W1 peptides, are likely to react also with self peptides so that they have a higher probability to be negatively selected. On the contrary antibodies targeting a peptide far from self, such as W4 peptides, have a very low probability to cross-react with self peptides, so that they have a smaller probability to be negatively selected and removed. This leads to a higher potential number of antibodies that can recognize peptides far from self with respect to peptides close to self.

Finally we studied the aminoacid frequencies for each of the nine positions in the sequence for the top ranking peptides. Logo plot for the most promising 100 peptides (selected considering the whole set TC) has been obtained by online tool WebLogo 3 [41].

**Fig 5:**
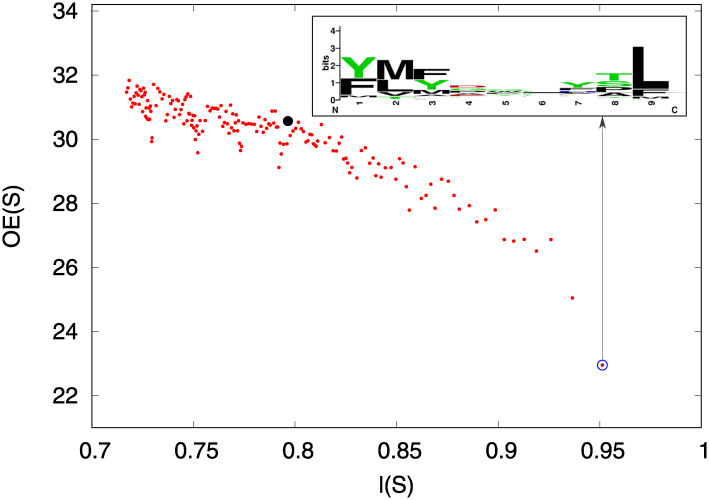
Overall entropy values, *OE*(*S*), for disjoint peptides set of Trypanosoma cruzi, *S*, (increasingly ordered according to the score *l*) plotted against the average score in the set, *l*(*S*). The black point is the entropy value associated to the 100 top ranking *W*_4_ peptides of Trypanosoma cruzi while the circled point at the bottom right represents the entropy value associated to the most promising 100 peptides selected from the whole set TC. In the associated inset it is shown the logo plot for this last set.

As can be observed in the inset of Fig 5 the selected 100 peptides show a pattern that is particularly evident in the initial (1 and 2) and terminal (9) positions of the 9-mers. We hypothesized a relationship between the score, *l*(*p*), and the presence of a pattern, in other words the higher the score, that is to say the higher the probability to be a potential epitope, the stronger the constraints on the peptide, especially in the initial and terminal positions.

This hypothesis was supported by an information content analysis. We considered the set of best ranking peptides increasingly ordered with respect to the score *l*(*p*) (ranging from 0.71 to 0.97, where 0.71 refers to the minimum score in the top hundred *W*_4_ peptides and 0.97 is the maximum score in TC) in total 17700 peptides that we divided in 177 groups, namely *S*_1_, *S*_2_, …, *S*_*k*_, …, *S*_177_, of 100 sequences according to their scores. We defined an overall entropy (*OE*) value for each set of 100 peptides, *S*_*k*_ (with *k* = 1…177) as the sum of the entropies of the frequency distribution of aminoacids for each position, formally:

$$\begin{array}{c} OE\left(S_{k}\right)=\sum_{i=1}^{9}\sum_{j=1}^{20}p_{j}^{i}log_{2}\left(p_{j}^{i}\right) \end{array}$$ (14)

where $p_{j}^{i}$ is the frequency of *j*-th aminoacid in the *i*-th position for the considered set *S*_*k*_.

In Fig 5 the overall entropy, *OE*(*S*), of each set is plotted as a function of its average score, *l*(*S*). As can be observed there is a clear dependency of the overall entropy of peptide sets on their average scores. We remind that the maximum entropy value for each position is *log*_2_(20)≈4.32 and consequently the maximum *OE* is ≈38.90. It is worth noting that the difference between *OE* related to the highest score set (around 23) and *OE* related to lower scores (*OE* in the range of 28 and 32) is extremely significant, indicating a strong consensus pattern for selected sequences.

Moreover, overall entropy values associated to the 100 top ranking *W*_4_ (we call this set $W_{4}^{h}$ with scores ranging from 0.71 to 0.96, black point Fig 5) is comparable to the overall entropy values associated to sets *S*_*k*_ providing that $l\left(S_{k}\right)\approx l\left(W_{4}^{h}\right)$. This property indicates that the information content is strictly linked to the average score of the set.

## 4 Conclusions and discussions

Recognition of potential pathogens attacking the human body is the first step to activate an immune response. Recognition is based on the ability of immune system to distinguish exogenous from endogenous peptides, according to the self/non-self paradigm. However, the mechanisms underlying the immune response show a high level of complexity that we still don’t completely understand. In this context, identification of potential epitopes, exogenous peptides able to trigger an immune response, is crucial in the view of designing new generation vaccines, following the strategy of reverse vaccinology.

In this work we analyzed the relationship between the distance from human self of given peptides and their probability to be presented on a cell surface, revealing higher value of that probability for peptides farther from human self. This kind of relationship is reasonable and coherent with the self/non-self paradigm but, as far as we know, it was never directly observed before. In particular peptides in the class *W*_4_ strongly bound a significantly higher number of HLAs with respect to peptides closer to human self. This result could be extremely favorable for the design of vaccines that would be suitable for large portion of population.

We studied the three steps leading a peptide to be presented on the cell surface, i.e., proteasome cleavage, TAP transport and MHC-I binding, revealing a significant strong co-evolution among them. A similar analysis was performed by Burroughs in 2004 [21] considering only two HLAs. Here we confirmed their results extending the analysis to all 81 available HLAs and we additionally showed that the observed co-evolution is even stronger when *W*_4_ peptides are considered.

Moreover, using the overall probability to be cleaved and the number of HLAs a peptide strongly binds, we identified two sets of promising epitopes, from TC and from *W*_4_, that are suitable for experimental tests to validate their immunogenicity.

An information content analysis performed on the set of 100 most promising peptides indicates an evident aminoacid pattern, reported through logo plot. Moreover the analysis, extended to a larger set of best ranking peptides, reveals that the overall entropy decreases as the average score increases.

In our opinion the identification of *W*_4_ peptides is extremely interesting in the view of vaccine design for several reasons. Firstly *W*_4_ peptides show a higher probability to be exposed and they can be presented on cell surface by a higher number of HLAs than expected. Secondly it can be hypothesized, due to a large cross-reactivity of *W*_4_ presented peptides, that they should have on average a higher number of potential antibodies able to recognize them and consequently trigger an immune response. Thirdly the use of *W*_4_ peptides as vaccine candidates avoids the risk of autoimmunity because of the low sequence similarity with human self.

Finally results obtained in this work can be read from two different points of view. The former is theoretical: the finding that the distance of a peptide from human self affects its likelihood to be an epitope can contribute to add new knowledge on the self/non-self paradigm. The latter is practical: those findings can provide a further selective criterion to epitope search in designing potential vaccines. Moreover, W4 peptides have at least three different amino acids from every self peptide, so if we align them with human protein sequences we will obtain a sequence identity always smaller than 66% in the blast best scores and this guarantees a very small probability of the induced antibody to cross-react with human self and to cause autoimmunity. As a case study we applied our methodology to *Trypanosoma cruzi*, the etiologic agent of Chagas disease, but the whole pipeline described in this work can be easily applied to any pathogen simply starting from its proteome. Nullomer peptide classes can be obtained through the free software available at http://www.iasi.cnr.it/~dsantoni/nullomers.
